# Real-World Outcomes of Revascularization Strategies in Patients With Left Ventricular Dysfunction and Three-Vessel Coronary Disease Stratified by Mitral Regurgitation

**DOI:** 10.3389/fcvm.2021.675722

**Published:** 2021-06-24

**Authors:** Qin Fan, Jun Liu, Yan Xu, Ruiqing Ni, Rui Xi, Fang Wang, Jian Hu, Hongyue Sun, Zhenkun Yang, Mi Zhou, Ruiyan Zhang, Qiang Zhao, Rong Tao

**Affiliations:** ^1^Department of Vascular and Cardiology, Rui Jin Hospital, Shanghai Jiao Tong University School of Medicine, Shanghai, China; ^2^Institute of Cardiovascular Diseases, Shanghai Jiao Tong University School of Medicine, Shanghai, China; ^3^Department of Cardiovascular Surgery, Rui Jin Hospital, Shanghai Jiao Tong University School of Medicine, Shanghai, China; ^4^Institute for Biomedical Engineering, ETH Zurich and University of Zurich, Zurich, Switzerland; ^5^University of Rochester, Rochester, New York, NY, United States

**Keywords:** ischemic cardiomyopathy, revascularization, mitral regurgitation, heart failure, percutaneous coronary intervention, coronary artery bypass grafting surgery

## Abstract

**Aims:** Limited information exists regarding optimal revascularization options for patients with triple-vessel coronary artery disease (TVD), heart failure (HF), and different degrees of mitral regurgitation (MR). Thus, we aimed to compare the effect of percutaneous coronary intervention (PCI) and coronary artery bypass grafting (CABG) surgery in the indicated patients.

**Methods and Results:** In the real-world prospective study, 1190 patients with multi-vessel disease and decreased left ventricular systolic function but without severe MR, who underwent PCI or CABG, were enrolled and followed-up for 4.7 ± 1.8 years. The primary endpoint was a composite of cardiovascular death and HF hospitalization. Secondary endpoints were the individual components of the primary outcome. Risk of the primary endpoint was higher in the PCI than in the CABG group (HR = 1.38, 95%CI: 1.14–1.67, and *P* < 0.01), particularly in patients with moderate MR (HR = 1.85, 95%CI: 1.35–2.55, and *P* < 0.01). In patients with no-mild MR, the risk of the primary endpoint did not differ significantly between PCI and CABG (*P* = 0.09). Treatment with PCI was associated with an increased risk for cardiovascular death and HF hospitalization in the moderate MR cohort, while PCI was comparable to CABG in the no-mild MR cohort.

**Conclusions:** In this real-world study, for patients with HF and TVD, CABG was related to lower adverse outcome rates compared to PCI. Assessment of MR can aid in selecting optimal revascularization therapies and in risk stratification.

## Introduction

Coronary artery disease (CAD) is the most common cause of left ventricular dysfunction (LVD), and its incidence has increased together with an increase in the associated mortality rates (1, 2). To date, many randomized trials and observational studies have shown that optimal revascularization, including coronary artery bypass surgery (CABG) and percutaneous coronary intervention (PCI), plays an important role in improving long-term survival and improving cardiac function in patients with LVD compared to medical treatment (3, 4). The advantages of CABG and PCI, especially for patients with different clinical features have also been compared in previous studies (3, 5, 6). However, the debate regarding the choice between CABG and PCI remains.

Current guidelines generally recommend CABG as the first-choice revascularization strategy for patients with triple-vessel disease (TVD) and heart failure (HF), while PCI should be considered as an alternative when complete revascularization can be achieved (2, 3). However, information is limited regarding the benefits of PCI in Chinese patients with systolic cardiac dysfunction. Further research exploring better revascularization strategy in patients with multiple comorbidities is also required (6).

Ischemic mitral regurgitation (IMR) is a common mechanical complication in patients with CAD, associated with poor prognosis (7). Nowadays, mitral valve surgery and concomitant CABG represent the most effective strategy for the treatment of severe symptomatic IMR (7, 8). However, in patients with mild to moderate IMR, mitral valve repair or replacement surgery at the time of CABG may not contribute to better survival or left ventricular (LV) reverse remodeling (9–11). These patients also benefitted from revascularization therapy evolving percutaneous therapies; however, there is no consensus on the accurate revascularization procedure (7). Therefore, the outcomes of CABG and PCI for patients with no to moderate IMR of different severities are not well-elucidated.

Previous studies have not evaluated “real-world” outcomes of patients with HF and TVD undergoing CABG vs. PCI in China, especially when stratified by IMR. Thus, our study aimed to fill in the gap by comparing the therapeutic effects of PCI and CABG on adverse events and prognosis in these representative subgroups. And we further examined whether IMR affects the results of different treatment strategies.

## Materials and Methods

### Study Design and Patients

We conducted a single-center prospective study designed to evaluate the treatment benefits of CABG vs. PCI for TVD combined with the presence of heart failure (NYHA II–IV) and systolic dysfunction [left ventricular ejection fraction (LVEF) ≤ 50%] in clinical practice. A total of 1,190 consecutive patients meeting the inclusion criteria underwent PCI or isolated CABG and discharged between December 1, 2012 and November 31, 2017 at Rui Jin Hospital (Shanghai, China) were included. TVD was defined as ≥70% stenosis in three coronary systems, namely, the left anterior descending (LAD) and diagonal arteries together with the left circumflex coronary artery (LCX) and obtuse marginal arteries, and the right coronary artery (RCA), with or without left main artery involvement (≥50% stenosis). Patients who had prior CABG or PCI, an acute myocardial infarction within 24 h before revascularization or presented with cardiogenic shock, and those who underwent concomitant valvular or aortic surgery, emergency or life-saving procedures were excluded. The exclusion criteria also included severe MR. At the time of enrollment, demographic data, including age, sex, and medical history were collected; blood pressure, heart rate, weight, and height were measured; and laboratory measurements and other medical examinations were also performed.

The study was approved by the institutional review committee of Rui Jin Hospital affiliated to Shanghai Jiao Tong University School of Medicine and conforms to the ethical guidelines of the 1975 Declaration of Helsinki. All patients gave written informed consent before screening and data collection.

### Study Procedures

PCI or CABG was selected according to a cardiac team including physicians, interventional cardiologists and cardiac surgeons, as well as patient's choice. All patients undergoing coronary arteriography were prescribed dual antiplatelet agents before invention. PCI was performed according to current practice guidelines, while the PCI strategy and stent type were left to the physician's discretion. CABG was performed using off-pump coronary artery bypass technique. The internal thoracic artery was preferentially used for LAD, and complete revascularization was performed using saphenous vein grafts. After the procedure, aspirin was continued indefinitely, and dual antiplatelet agents were recommended for about 12 months. Optimal medication for HF and CAD, including renin-angiotensin-aldosterone system inhibitors, β-blockers, and statins were routinely used during the study period.

Echocardiographic measurements were performed by experienced cardiac sonographers and were interpreted by board-certified cardiologists. LVEF was assessed using biplanar Simpson's method. Mild mitral regurgitation was defined as an effective regurgitant orifice area (EROA) <0.2 cm^2^, regurgitant volume <30 ml/beat, and Doppler vena contracta width <0.3 cm, whereas severe MR included EROA ≥ 0.4 cm^2^, regurgitant volume >60 ml/beat, or Doppler vena contracta width ≥0.7 cm. The patients with indices between the mild and severe comprised the moderate group.

### Outcomes and Clinical Follow-Up

The primary endpoint was defined as a composite of cardiovascular death and first re-admission due to HF. And the secondary endpoints were cardiovascular death and HF hospitalization, respectively. The diagnosis of recurrent heart failure was based on clinical symptoms, physical signs, elevated laboratory biomarkers or radiological evidence of pulmonary congestion. All outcomes were intensively verified and adjudicated by independent clinicians. Clinical follow-up was performed by survey via telephone or hospital visit.

### Statistical Analysis

Baseline continuous variables were expressed as the mean ± standard deviation (SD) and compared using the Student's *t*-test, while categorical variables were presented as numbers with percentage and compared between the two groups using Chi-square tests. Baseline characteristics were compared between the PCI and CABG group. Kaplan–Meier analysis and log-rank tests were performed to describe and compare the survival curves for the two procedures for each endpoint. The hazard ratios (HR) of PCI compared to those of CABG were further assessed using univariate and multivariable Cox proportional hazards regression models, adjusted for influential factors including age, sex, body mass index (BMI), concomitant conditions, such as hypertension, diabetes mellitus (DM) and dyslipidemia, levels of creatinine, NT-pro brain natriuretic peptide (BNP), glycated hemoglobin (HbA1c), low-density lipoprotein(LDL) and D-dimer, performed and expressed with 95% confidence intervals. Consistency of treatment effects in different subgroups was assessed by cox regression models with tests for interaction. Patients who were lost to follow-up were calculated according to censored data. All *P*-values were 2-sided, and *P* < 0.05 were considered statistically significant. The SPSS software, version 22.0 was used for the statistical analyses. The investigators had full access to the data and took responsibility for maintaining data integrity.

## Results

### Patients' Characteristics

**Figure 1:**
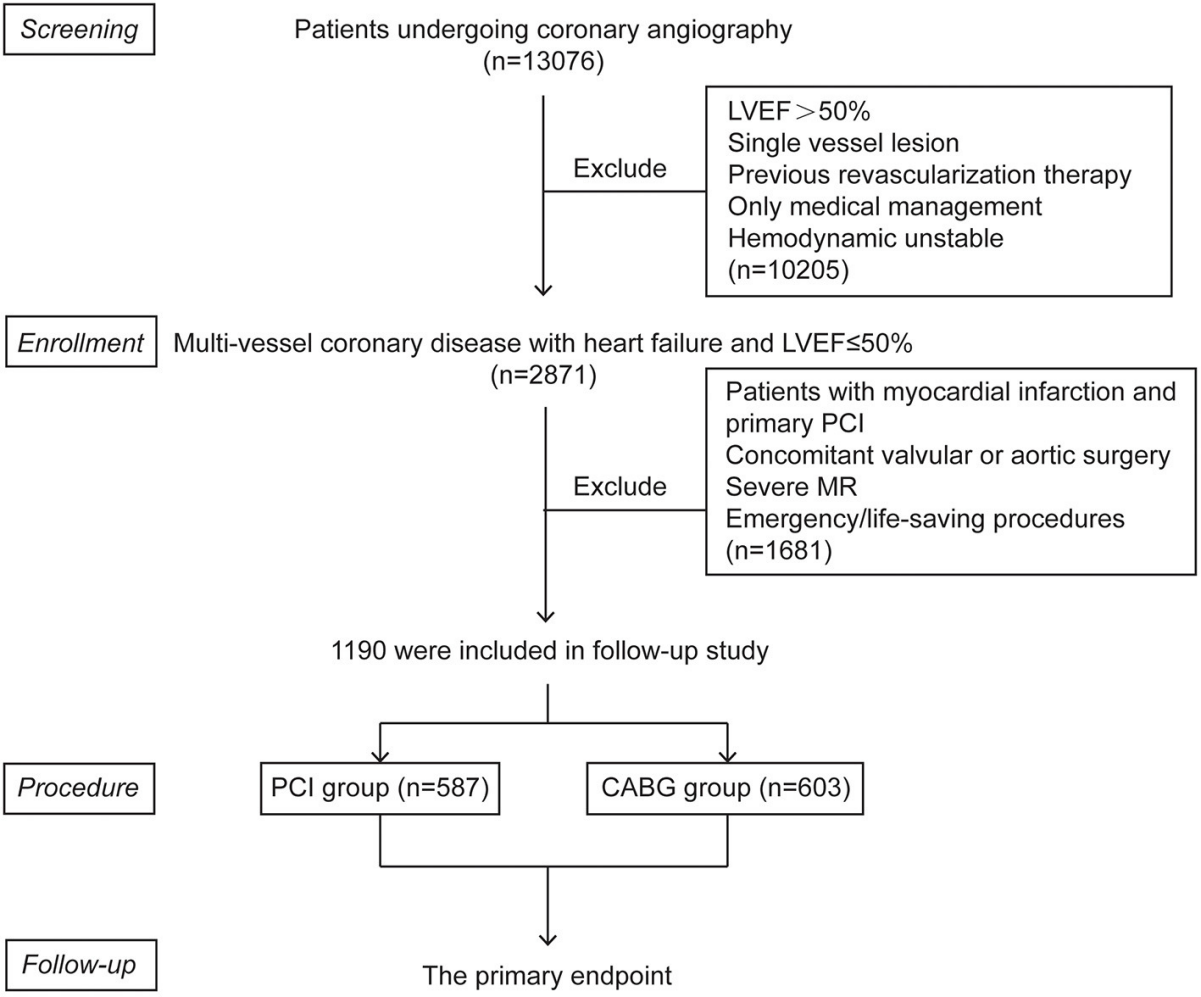
Patient flow chart for the study cohort. CABG, coronary artery bypasses grafting; LVEF, left ventricular ejection fraction; MR, mitral regurgitation; PCI, percutaneous coronary intervention.

A total of 1,190 consecutive patients with TVD and LVD were included in the study. Among these patients, 587 (49.3%) were in the PCI group and 603 (50.7%) were in the CABG group (Figure 1). The mean age was 64.64 ± 10.65 years and 1,025 (86.1%) were men. Baseline characteristics are summarized in Table 1. As illustrated, patients who underwent CABG had lower levels of hemoglobin, LDL and LVEF. The demographic characteristics, comorbidities, NTproBNP levels, NYHA classifications, coronary artery SYNTAX score, and medication at discharge were comparable between these two groups.

**Table 1:** Baseline characteristics of study population by treatment strategies.

	**CABG (*n* = 603)**	**PCI (*n* = 587)**	***P*-value**
**Demographic characteristics**			
Age (years)	64.36 ± 9.49	64.91 ± 11.73	0.38
Male	521 (86.4)	504 (85.9)	0.79
Current Smoking	251 (41.6)	210 (35.8)	0.04
Current Drinking	102 (16.9)	122 (20.8)	0.09
Body mass index (kg/m^2^)	24.51 ± 3.27	24.51 ± 3.29	0.98
Systolic blood pressure (mmHg)	127.02 ± 20.06	127.84 ± 22.25	0.5
Diastolic blood pressure (mmHg)	72.67 ± 12.09	74.99 ± 13.48	<0.01
Heart rate (beats/min)	78.52 ± 13.31	81.53 ± 14.30	<0.01
Family history	73 (12.1)	84 (14.3)	0.26
**Medical history**			
Atrial fibrillation	51 (8.5)	50 (8.5)	0.97
Hypertension	422 (70.0)	411 (70.0)	0.99
Diabetes mellitus	271 (44.9)	252 (42.9)	0.49
Dyslipidemia	268 (44.4)	231 (39.4)	0.08
Renal dysfuncion	68 (11.3)	83 (14.1)	0.14
**NYHA classification**			0.50
II	266 (44.1)	276 (47.0)	
III	307 (50.9)	279 (47.5)	
IV	30 (5.0)	32 (5.5)	
**Lab. examination**			
Hemoglobin (g/L)	129.31 ± 15.97	132.16 ± 17.06	<0.01
Platelet (*10^9/L^)	194.05 ± 65.18	191.68 ± 64.49	0.53
HbA1c (%)	6.79 ± 1.49	6.73 ± 1.55	0.54
Alanine aminotransferase (IU/L)	36.92 ± 149.89	38.42 ± 45.31	0.82
Albumin (g/L)	35.95 ± 4.31	35.58 ± 4.63	0.16
Creatinine (μmol/L)	95.46 ± 35.54	96.22 ± 65.97	0.81
Uric acid (μmol/L)	370.32 ± 105.46	361.29 ± 107.21	0.14
LDL-C (mmol/L)	2.37 ± 0.93	2.54 ± 0.98	<0.01
NTproBNP (pg/ml)	2,588.60 ± 3,302.63	2,602.02 ± 3,184.66	0.94
D-Dimer (mg/L)	0.82 ± 2.34	0.82 ± 1.56	0.99
LVEF (%)	41.02 ± 6.79	42.36 ± 6.70	<0.01
LAd (mm)	43.13 ± 4.71	41.96 ± 4.89	<0.01
LVEDd (mm)	59.22 ± 6.14	56.75 ± 6.80	<0.01
LVESd (mm)	45.95 ± 6.45	43.51 ± 7.08	<0.01
No-mild MR	424 (70.3)	435 (74.1)	0.15
SYNTAX score	22.72 ± 6.05	22.36 ± 5.69	0.29
**Medications**			
ACEI/ARB/ARNI	447 (74.1)	455 (77.5)	0.17
β-blocker	537 (89.1)	503 (85.7)	0.08
Statins	572 (94.9)	558 (95.1)	0.87
Hypoglycemic drugs	155 (25.7)	164 (27.9)	0.38

*Data are presented as the mean ± SD or n%*.

*ACEI, angiotensin-converting enzyme inhibitor; ARB, angiotensin receptor blocker; ARNI, dual angiotensin-receptor/ NEP inhibitors; HbA1c, glycated hemoglobin; LAd, left atrium diameter; LDL, low-density lipoprotein; LVEF, left ventricular ejection fraction; LVEDd, left ventricular end-diastolic diameter; LVESd, left ventricular end-systolic diameter; NT-proBNP, pro-brain natriuretic peptide; NYHA, New York Heart Association*.

### Outcome According to the Therapeutic Strategies

The mean follow-up was 4.7 ± 1.8 years for the overall cohort, with 99.3% patients completing the follow-up for all endpoints obtained. During the follow-up, a total of 439 (36.9%) patients met the primary endpoint. With respect to the secondary endpoints, 189 (15.9%) patients died of a cardiovascular cause, and 281 (23.6%) patients had been hospitalized because of HF.

**Figure 2:**
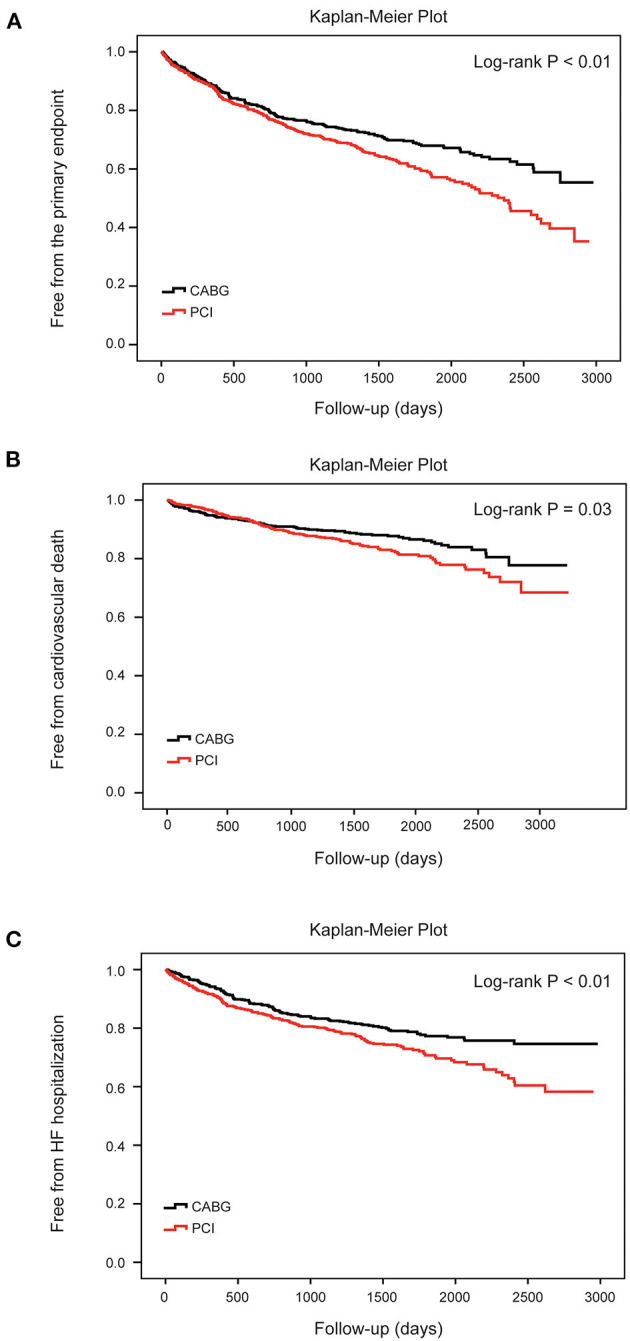
Cumulative risks for the primary and secondary endpoints in all patients. Kaplan–Meier survival curves for the primary endpoint **(A)** (log-rank *P* < 0.01), cardiovascular death **(B)** (log-rank *P* = 0.03) and heart failure hospitalization **(C)** (log-rank *P* < 0.01) according to different therapy strategies in all patients. Differences between the two procedures were evaluated by the log-rank test. CABG, coronary artery bypasses grafting; HF, heart failure; PCI, percutaneous coronary intervention.

During the follow-up, the frequency of the primary endpoint was significantly lower in patients who underwent CABG than in those who underwent PCI, as indicated by the observed event-free Kaplan-Meier survival curves and log-rank analyses (32.3 vs. 41.6%, *P* < 0.01; Figure 2A). Multivariable Cox proportional hazard analyses identified that the risk of the primary endpoint was significantly higher in the PCI group after adjusting for age, sex, and conventional risk factors (HR = 1.38, 95%CI: 1.14–1.67, and *P* < 0.01; Table 2). PCI was also associated with a significantly higher risk for cardiovascular death (18.2 vs. 13.6%, log-rank *P* = 0.03) and HF hospitalization (27.1 vs. 20.2%, log-rank *P* < 0.01) compared with CABG, respectively (Figures 2B,C). The difference remained significant after adjusting for influential factors in the Cox proportional hazard analysis (Table 2).

**Table 2:** Cox proportional hazard models for clinical outcomes after PCI as compared with after CABG in all patients.

	**Unadjusted HR (95% CI)**	***P-*value**	**Adjusted HR (95% CI)**	***P-*value**
	**PCI:CABG**		**PCI:CABG**	
Primary endpoint	1.37 (1.14–1.66)	<0.01	1.38 (1.14–1.67)	<0.01
Cardiovascular death	1.38 (1.03–1.84)	0.03	1.34 (1.00–1.79)	0.05
HF hospitalization	1.42 (1.12–1.79)	<0.01	1.45 (1.15–1.84)	<0.01

*HR was adjusted for age, sex, body mass index, history of hypertension, history of DM, history of dyslipidemia, creatinine, HbA1c, LDL, D-dimer and NT-proBNP; CABG, coronary artery bypass grafting; CI, confidence interval; DM, diabetes mellitus; HbA1c, glycated hemoglobin; HF, heart failure; HR, hazard ratio; LDL, low-density lipoprotein; NT-proBNP, pro-brain natriuretic peptide; PCI, percutaneous transluminal coronary intervention*.

### Subgroup Analysis Between PCI and CABG for the Primary Endpoint

**Figure 3:**
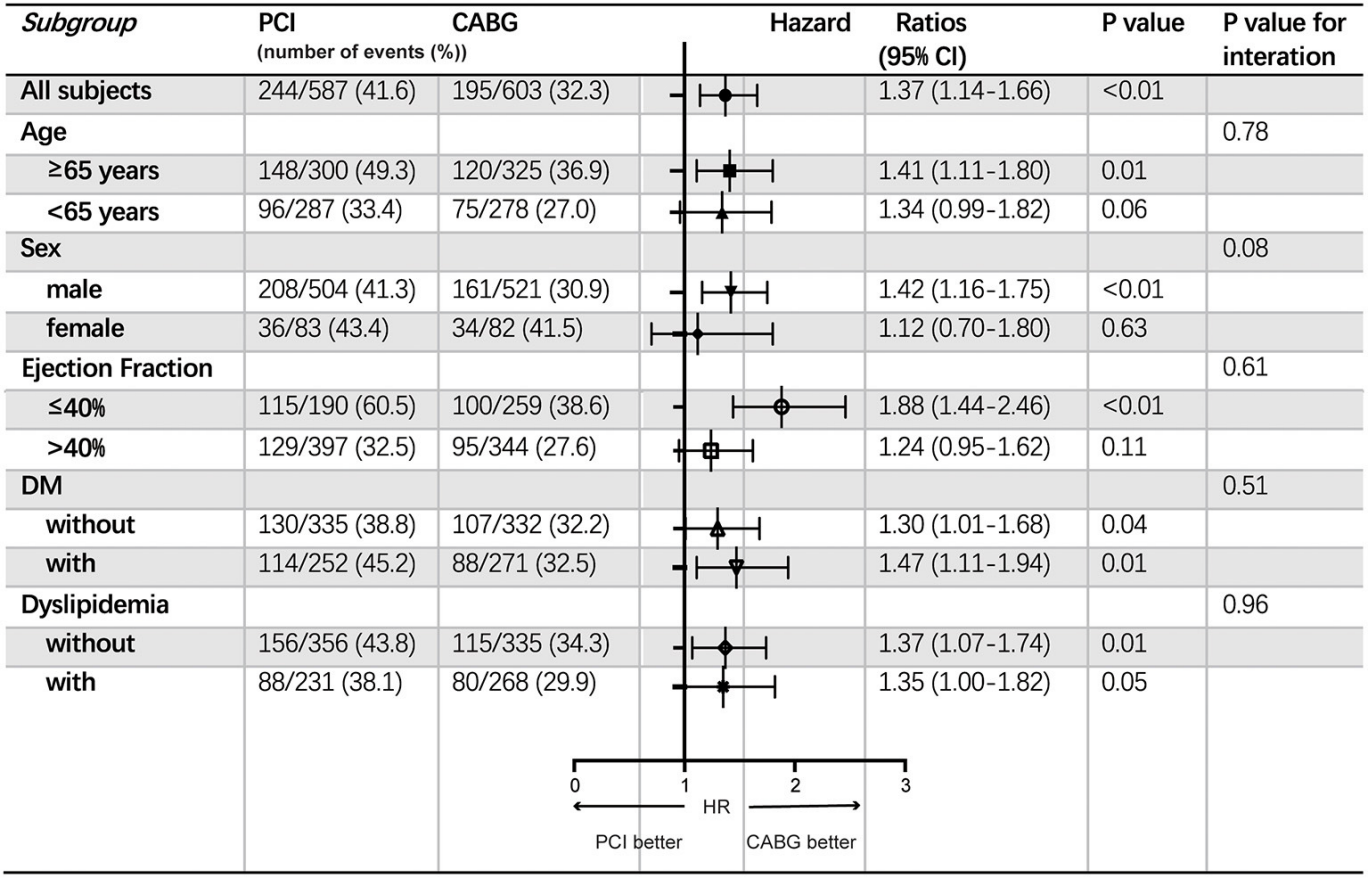
Subgroup analyses of the primary endpoint. Event rates and hazard ratios with 95% confidence intervals are shown for the primary endpoint in all patients stratified into different subgroups according to age, sex, ejection fraction and comorbidities including DM and dyslipidemia. CABG, coronary artery bypasses grafting; CI, confidence interval; DM, diabetes mellitus; HR, hazard ratio; PCI, percutaneous coronary intervention.

The increased risk of the primary endpoint after PCI compared with CABG treatment was more remarkable in male patients older than 65 with EF ≤ 40%, according to subgroup analysis. Nevertheless, common comorbidity such as DM or dyslipidemia did not affect the difference of the incidence of the primary endpoint between patients with CABG and PCI (Figure 3).

### Effect of the Mitral Regurgitation Severity

We further compared the event rates between the two procedures in all patients stratified by the severity of MR at baseline. Patients with moderate MR had lower LVEF (39.53 ± 6.94% vs. 42.51 ± 6.53%, *P* < 0.01) and higher levels of NT-proBNP (3,518.64 ± 3,454.20 vs. 2,239.40 ± 3,087.90, and *P* < 0.01) as compared to those with no-mild MR. In patients with no-mild MR, the SYNTAX score was 22.42 ± 5.74, while in those with moderate MR, the SYNTAX score was 22.86 ± 6.20 (*P* = 0.24).

**Figure 4:**
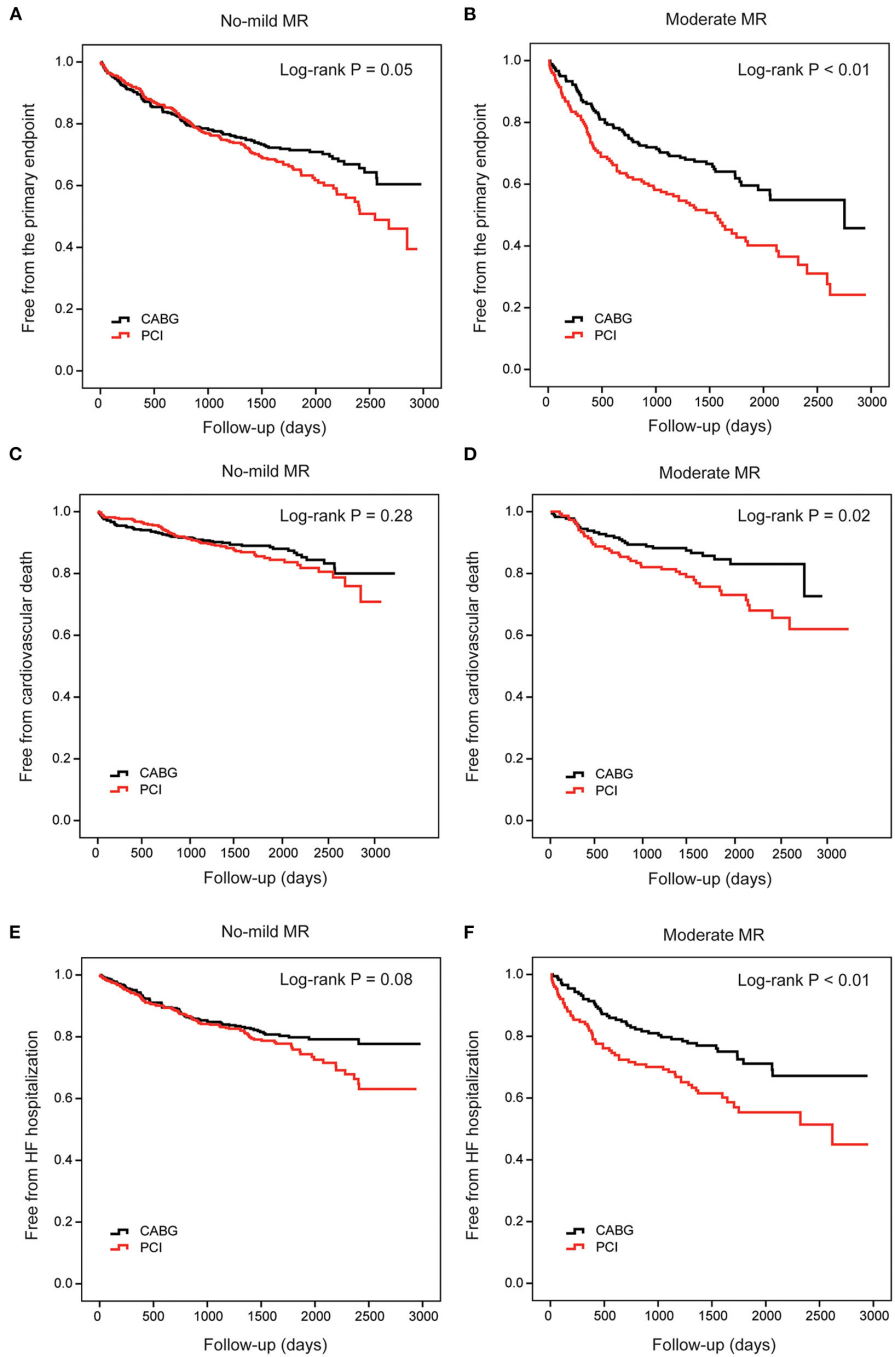
Cumulative risks for the primary and secondary endpoints in patients stratified by MR. Kaplan–Meier survival curves of the primary endpoint in patients with no-mild MR **(A)** and moderate MR **(B)** are compared between percutaneous coronary intervention (PCI) and coronary artery bypasses grafting (CABG), respectively. Survival curves of cardiovascular death **(C,D)** and heart failure hospitalization **(E,F)** are also compared between PCI and CABG in these two groups, respectively. CABG, coronary artery bypasses grafting; HF, heart failure; MR, mitral regurgitation; PCI, percutaneous coronary intervention.

During the follow-up, in patients with no-mild MR, the cumulative incidence of the primary endpoint was different between PCI and CABG (35.9 vs. 29.5%, log-rank *P* = 0.05; Figure 4A). However, the risk did not differ significantly after full adjustment in the Cox proportional hazard model (HR: 1.23, 95% CI: 0.97–1.56, and *P* =0.09; Table 3). In patients with moderate MR, frequencies of the primary endpoint for PCI were significantly increased compared with CABG treatment (57.9 vs. 39.1%, log-rank *P* < 0.01; Figure 4B). And PCI was associated with a significantly higher risk vs. CABG after full adjustment (HR: 1.85, 95% CI: 1.35–2.55, and *P* < 0.01; Table 3).

**Table 3:** Risk of the primary and secondary endpoints in patients stratified by the severity of MR.

	**Unadjusted HR (95% CI)**	***P*-value**	**Adjusted HR (95% CI)**	***P*-value**
	**PCI:CABG**		**PCI:CABG**	
**Primary endpoint**				
No-mild MR	1.27 (1.00–1.61)	0.05	1.23 (0.97–1.56)	0.09
Moderate MR	1.73 (1.27–2.37)	<0.01	1.85 (1.35–2.55)	<0.01
**Cardiovascular death**				
No-mild MR	1.22 (0.85–1.75)	0.28	1.06 (0.73–1.52)	0.78
Moderate MR	1.82 (1.12–2.96)	0.02	2.03 (1.22–3.38)	0.01
**HF hospitalization**				
No-mild MR	1.30 (0.97–1.76)	0.08	1.32 (0.98–1.77)	0.07
Moderate MR	1.80 (1.22–2.66)	<0.01	1.86 (1.25–2.76)	<0.01

*HR was adjusted for age, sex, body mass index, history of hypertension, history of DM, history of dyslipidemia, creatinine, HbA1c, LDL, D-dimer and NT-proBNP; CABG, coronary artery bypass grafting; CI, confidence interval; DM, diabetes mellitus; HbA1c, glycated hemoglobin; HF, heart failure; HR, hazard ratio; LDL, low-density lipoprotein; MR, mitral regurgitation; NT-proBNP, pro-brain natriuretic peptide; PCI, percutaneous transluminal coronary intervention*.

When cardiovascular death alone was the clinical endpoint, the event rates and cumulative incidence were statistically similar for PCI and CABG in patients with no-mild MR (Figure 4C). However, in patients with moderate MR, the mortality rate due to cardiovascular disease was higher for PCI (Figure 4D). Cox proportional hazard analysis further demonstrated that PCI was associated with a remarkably higher risk for cardiovascular death compared with CABG only in patients with moderate MR (HR: 2.03, 95% CI: 1.22–3.38, and *P* = 0.01; Table 3).

Similarly, when HF hospitalization was the clinical endpoint, the cumulative incidence was also comparable between PCI and CABG for patients with no-mild MR (Figure 4E); while for those with moderate MR, the difference was significant (Figure 4F). The adjusted risk for HF hospitalization was significantly higher after PCI in patients with moderate MR (HR: 1.86, 95% CI: 1.25–2.76, *P* < 0.01); however, in those with no-mild MR, the risk did not show a remarkable increase (Table 3).

### Subgroup Analysis Between PCI and CABG Stratified by the Severity of Mitral Regurgitation

In subgroup analysis, the increased risk for the primary endpoint in patients with PCI vs. CABG was more remarkable in those with moderate MR, compared to those with no-mild MR, especially in male patients older than 65 years old. Furthermore, the patients with EF > 40%, PCI was comparable with CABG in the no-mild MR group, while PCI led to higher risk in the moderate group. However, in patients with EF ≤ 40% at baseline, PCI was associated with a higher risk for the primary endpoint compared with CABG in both patients with no-mild MR and with moderate MR. The comorbidity of DM or dyslipidemia did not affect the difference from no-mild MR group to moderate MR group regarding to the comparison between CABG and PCI (Table 4).

**Table 4:** Subgroup analyses of the primary endpoint in patients stratified by the severity of MR.

	**PCI**	**CABG**	**HR (95% CI)**	***P-*value**	***P-*value for interaction**
	**Number of events (%)**		**PCI:CABG**		
**Age** **≥** **65 years**					<0.01
No-mild MR	89/204 (43.6)	75/213 (35.2)	1.22 (0.90–1.66)	0.21	
Moderate MR	59/96 (61.5)	45/112 (40.2)	1.94 (1.32–2.87)	<0.01	
**Age** **<** **65 years**					<0.01
No-mild MR	67/231 (29.0)	50/211 (23.7)	1.35 (0.93–1.94)	0.11	
Moderate MR	29/56 (51.8)	25/67 (37.3)	1.41 (0.83–2.41)	0.21	
**Male**					<0.01
No-mild MR	138/384 (35.9)	107/380 (28.2)	1.35 (1.05–1.73)	0.02	
Moderate MR	70/120 (58.3)	54/141 (38.3)	1.76 (1.23–2.51)	<0.01	
**Female**					0.25
No-mild MR	18/51 (32.3)	18/44 (40.9)	0.83 (0.43–1.61)	0.83	
Moderate MR	18/32 (56.3)	16/38 (42.1)	1.61 (0.82–3.16)	1.61	
**EF** **≤** **40%**					<0.01
No-mild MR	61/114 (53.5)	56/164 (34.1)	1.82 (1.27–2.62)	<0.01	
Moderate MR	54/76 (71.1)	44/95 (46.3)	1.96 (1.31–2.91)	<0.01	
**EF** **>** **40%**					0.01
No-mild MR	95/321 (29.6)	69/260 (26.5)	1.15 (0.84–1.57)	0.38	
Moderate MR	34/76 (44.7)	26/84 (31.0)	1.69 (1.01–2.82)	0.05	
**Without DM**					<0.01
No-mild MR	82/248 (33.1)	70/229 (30.6)	1.16 (0.84–1.59)	0.38	
Moderate MR	48/87 (55.2)	37/103 (35.9)	1.80 (1.17–2.76)	0.01	
**With DM**					<0.01
No-mild MR	74/187 (39.6)	55/195 (28.2)	1.42 (1.00–2.01)	0.05	
Moderate MR	40/65 (61.5)	33/76 (43.4)	1.65 (1.04–2.61)	0.03	
**Without dyslipidemia**					<0.01
No-mild MR	97/257 (37.7)	68/229 (29.7)	1.34 (0.98–1.82)	0.07	
Moderate MR	59/99 (59.6)	47/106 (44.3)	1.58 (1.07–2.31)	0.02	
**With dyslipidemia**					<0.01
No-mild MR	59/178 (33.1)	57/195 (29.2)	1.17 (0.81–1.69)	0.4	
Moderate MR	29/53 (54.7)	23/73 (31.5)	1.90 (1.10–3.30)	0.02	

*CABG, coronary artery bypass grafting; CI, confidence interval; DM, diabetes mellitus; EF, ejection fraction; HR, hazard ratio; MR, mitral regurgitation; PCI, percutaneous transluminal coronary intervention*.

## Discussion

The present study investigated the long-term clinical outcomes of PCI vs. CABG in Chinese patients with three-vessel stenosis and LV systolic dysfunction. We indicated that the rate of cardiovascular death and HF hospitalization was lower in the CABG group than in PCI. Such difference of frequency between PCI and CABG was more remarkable in patients with moderate MR than in those with no-mild MR (18.8% difference with PCI and CABG in moderate MR patients vs. 6.4% difference with PCI and CABG in no-mild MR patients). Assessment of MR could help risk stratification and decision-making in such kinds of patients when considering optimal revascularization therapy.

First, among patients with multi-vessel coronary disease, several clinical trials, for example, SYNTAX, FREEDOM, and EXCEL consistently showed early and sustained improvement in survival for both PCI and CABG during long-term follow-up (12–15). However, the results of comparison between CABG and PCI were controversial, and patients with HF were largely excluded; therefore, the available data of these patients are insufficient.

The present study indicated that CABG could more remarkably reduce the adverse outcomes, compared to PCI, in TVD patients with LV systolic dysfunction, which supported the results of several studies evaluating revascularization therapy (16, 17), and also supplemented the evidence of current guidelines especially in Chinese patients.

It has been demonstrated that the second-generation DES that is widely used today could improve efficacy, safety, and device performance, showing similar risk of death and long-term clinical outcomes compared with CABG (18, 19). However, our study found that CABG was superior to second-generation DES among selected patients with multi-vessel disease and LVD. One of the underlying reasons may be that CABG could contribute to more complete revascularization compared with multi-vessel PCI, since micro-vascular ischemia might be persistent, and <70% stenosis was not treated with PCI (20). It is also reasonable that restenosis would occur more easily and show a more negative impact in HF patients due to more complex and diffuse coronary artery stenosis. Moreover, usually PCI procedure is of longer duration in those with complex CAD and HF, together with possibly impaired kidney function accompanied by HF, thus potentially contributing to a higher risk of renal failure and mortality rates (21).

Secondly, we found that in patients with TVD, LVD and moderate MR, CABG was related to better long-term survival vs. PCI; however, the difference was non-significant in those with no-mild MR. Recent guidelines state that the choice of revascularization in patients with ICM is best based on clinical features, for instance, clinical statue, coronary anatomy, magnitude of systolic dysfunction, comorbidities, patient preferences, as well as consultant and judgment between the interventional cardiologist and the cardiac surgeon (3, 22). The coexisting valvular disease should especially be carefully evaluated. Nevertheless, previous studies comparing CABG vs. PCI in the indicated ICM patients did not consider MR (23). Our findings added to existing literature that the degree of MR before revascularization influenced the effectiveness of CABG and PCI, expanding current evidence before decision-making. Therefore, PCI may be an acceptable alternative to CABG in ICM patients with no-mild MR, relatively higher LVEF, and in whom complete revascularization can be achieved. However, in other patients, especially in those with moderate MR, CABG remained more effective.

The most common initial insult in patients with chronic IMR is remodeling of the LV following ischemia (7). Once IMR occurs, it progresses as changes in LV size and shape, which results in worsening cardiac function (24). One potential interpretation of the finding is that MR might be a marker for systolic dysfunction that might respond better to CABG. On the other hand, although the stenosis of main coronary branches was similar, patients with moderate MR are likely to show more severe myocardial ischemia of diffuse small vessels than those without MR and could benefit more from CABG because of complete revascularization including the small vessels and capillaries which could not be treated by PCI.

Furthermore, in the present study, dual antiplatelet agents were recommended for about 12 months after the procedure and aspirin was continued indefinitely. However, patients who received revascularization therapy again during the follow-up might continue to have dual antiplatelet treatment for some time, which may influence the prognosis. Future study will be needed to explore the incidence of re-revascularization therapy, and the cumulative duration of dual antiplatelet treatment of CABG and PCI in these patients.

In addition, since patients with severe MR preferred to have concomitant mitral valve repair or replacement surgery with CABG (25, 26), which would affect the comparison, they were not included in the present study. However, whether those with moderate IMR should take valve surgery or not is debatable. A randomized trial on treatment of moderate IMR showed that addition of surgical mitral valve repair to CABG made no significant difference to survival or LV reverse remodeling at 2 years (9, 10). We also found that these patients could benefit from isolated CABG. However, the comparison between the valve surgery and CABG alone needs to be analyzed in future studies. Besides, taking other influential factors especially NT-proBNP levels and other biomarkers together with MR could help risk stratification to choose CABG or PCI for each patient precisely.

## Study Limitations

We chose to perform a real-world cohort study, which represents a more accurate account of clinical care. However, the present study had several limitations. First, since it was a non-randomized single-center observational study, unknown confounders may have affected the result. We hope to employ better designed randomized study in the future. Second, since the present study did not perform echocardiographic measurements of each patient at specific times after revascularization during the follow-up, the contribution of CABG and PCI on the development of MR and cardiac function in patients with different degree of MR at baseline needs to be further analyzed in long-term future studies. More parameters besides LVEF derived from echocardiography, such as global myocardial longitudinal strain rate and cardiac magnetic resonance imaging, might be assessed in the future to assess cardiac function. The specific values about the severity of MR including EORA and regurgitant volume, as well as the values evaluating the degree of ischemia such as FFR and scintigraphy will be obtained in future studies to evaluate the effect of different therapies on MR, revascularization and LV remodeling. In addition, the study included patients with atrial fibrillation. Since atrial MR as well as degenerative mitral insufficiency could play an important role in the process of worsening and recovery of HF, more specific values to differentiate different MR types need to be analyzed in future studies. Moreover, further studies exploring the influential factors or comorbidities leading to the use of specific revascularization procedures in cases of complicated CAD and LVD are required to devise a more effective selection strategy.

## Conclusion

Our study emphasizes the survival benefits and reduced risk for HF hospitalization with CABG compared to PCI in patients with TVD and systolic LVD, especially in those with moderate MR before the procedure. The degree of MR, together with other influential factors, could aid in decision-making while selecting between PCI and CABG for optimal revascularization therapy and risk stratification for these patients.

## Data Availability Statement

The raw data supporting the conclusions of this article will be made available by the authors, without undue reservation.

## Ethics Statement

The studies involving human participants were reviewed and approved by the institutional review committee of Rui Jin Hospital affiliated to Shanghai Jiao Tong University School of Medicine. The patients/participants provided their written informed consent to participate in this study.

## Author Contributions

QF, JL, RZ, QZ, and RT contributed to all stages including conception and design of the study, as well as acquisition, analysis, and interpretation of the data. QF contributed to drafting the article, together with RZ, QZ, and RT participating in revising and final approval of the version to be submitted. RN contributed to the interpretation of the data as well as to revising the manuscript. YX, RX, FW, and JH were integral to the design of the study as well as data collection. ZY, HS, and MZ assisted with data analysis and interpretation of the data. All co-authors assisted with revising the manuscript for important intellectual content and they also have read and approved the manuscript for submission. All authors made substantial contributions to the work presented in the paper and to the preparation of the paper itself.

## Conflict of Interest

The authors declare that the research was conducted in the absence of any commercial or financial relationships that could be construed as a potential conflict of interest.
